# Use of Physical Activity and Exercise to Reduce Inflammation in Children and Adolescents with Obesity

**DOI:** 10.3390/ijerph19116908

**Published:** 2022-06-05

**Authors:** Valeria Calcaterra, Matteo Vandoni, Virginia Rossi, Clarissa Berardo, Roberta Grazi, Erika Cordaro, Valeria Tranfaglia, Vittoria Carnevale Pellino, Cristina Cereda, Gianvincenzo Zuccotti

**Affiliations:** 1 Pediatric and Adolescent Unit, Department of Internal Medicine, University of Pavia, 27100 Pavia, Italy; erika.cordaro01@universitadipavia.it; 2 Pediatric Department, “Vittore Buzzi” Children’s Hospital, 20154 Milan, Italy; virginia.rossi@unimi.it (V.R.); roberta.grazi@unimi.it (R.G.); valeria.tranfaglia@unimi.it (V.T.); gianvincenzo.zuccotti@unimi.it (G.Z.); 3 Laboratory of Adapted Motor Activity (LAMA), Department of Public health, Experimental Medicine and Forensic Science, University of Pavia, 27100 Pavia, Italy; matteo.vandoni@unipv.it; 4 Department of Biomedical and Clinical Science, Università degli Studi di Milano, 20157 Milan, Italy; clarissa.berardo@unimi.it (C.B.); vittoria.carnevalepellino@unipv.it (V.C.P.); 5 Department of Industrial Engineering, University of Tor Vergata Rome, 00133 Rome, Italy; 6 Neonatal Screening and Metabolic Disorders Unit, V. Buzzi Children’s Hospital, 20154 Milan, Italy; cristina.cereda@asst-fbf-sacco.it

**Keywords:** inflammation, obesity, exercise, physical activity, children, adolescents

## Abstract

Childhood obesity is a leading public health problem worldwide, as it is increasingly prevalent and therefore responsible for serious obesity-related comorbidities, not only in childhood but also in adulthood. In addition to cardio-metabolic obesity-related disorders, recent evidence suggests that excess adipose tissue in turn is associated with immune cell infiltration, increased adipokine release, and the development of low-grade systemic inflammation obesity. Exercise is considered a non-pharmacological intervention that can delay obesity-related comorbidities, improving cardiovascular fitness and modulating the inflammatory processes. It has been reported that the anti-inflammatory effect of regular exercise may be mediated by a reduction in visceral fat mass, with a subsequent decrease in the release of adipokines from adipose tissue (AT) and/or by the induction of an anti-inflammatory environment. In this narrative review, we discuss the role of AT as an endocrine organ associated with chronic inflammation and its role in obesity-related complications, focusing on the effect of exercise in reducing inflammation in children and adolescents with obesity. Regular physical exercise must be considered as a natural part of a healthy lifestyle, and promoting physical activity starting from childhood is useful to limit the negative effects of obesity on health. The crucial role of the immune system in the development of obesity-induced inflammatory processes and the efficacy of exercise as an anti-inflammatory, non-pharmacological intervention may provide possible targets for the development of new treatments and early preventive strategies.

## 1. Introduction

Childhood obesity is a leading public health problem worldwide, as it is increasingly prevalent and therefore responsible for serious obesity-related comorbidities, not only in childhood but also in adulthood [1,2,3,4]. Childhood obesity is associated with increased comorbidities, including insulin resistance, diabetes mellitus, dyslipidemia, hypertension, and sleep apnea, which increase the risk of cardiovascular diseases and premature death during adulthood [1]. In addition to cardio-metabolic obesity-related disorders, recent evidence suggests that excess adipose tissue in turn is associated with immune cell infiltration, increased adipokine release, and the development of low-grade systemic inflammation obesity from pediatric to adult age [1].

An inactive lifestyle leads to the accumulation of adipose tissue, as energy expenditure is lower than energy intake.

Physical activity (PA) can be defined as any movement of the body that requires increased energy expenditure above the resting level. Exercise is part of PA and refers to planned and structured activity that aims to improve a specific outcome such as cardiorespiratory fitness, functional ability, body mass reduction, or body recompositing [5]. 

As recommended by the World Health Organization (WHO) in 2020, promoting an active lifestyle and appropriate levels of physical exercise is mandatory to reduce the risk of preventable adverse health events for all people [4]. Exercise is considered a non-pharmacological intervention that can delay obesity-related comorbidities, improving cardiovascular fitness and modulating inflammatory processes in both children and adults [6]. As reported by Gleeson, the anti-inflammatory effect of regular exercise may be mediated by a reduction in visceral fat mass, with a subsequent decreased release of adipokines from AT and/or by the induction of an anti-inflammatory environment [7]. 

In this narrative review, we discuss the role of AT as an endocrine organ associated with chronic inflammation and its role in obesity-related complications, focusing on the effect of exercise in reducing inflammation in children and adolescents with obesity. The crucial role of the immune system on the development of obesity-induced inflammatory processes and the efficacy of exercise as anti-inflammatory non-pharmacological intervention may provide possible targets for the development of new treatments and early preventive strategies. Considering that the acquisition of a healthy lifestyle could track to later ages, studying the phenomenon starting at childhood could offer a better preventive strategy to preserve children’s health.

## 2. Methods

A narrative review was performed; we presented a non-systematic summation and critical analysis of current knowledge on the topic of the role of exercise in the reduction in obesity-related inflammation, also considering data on adults [8]. To refine the scope of our narrative review, we established a set of inclusion criteria: articles published up to April 2022 in the English language; original scientific papers, clinical trials, meta-analyses, and reviews published on a specific topic; and articles on pediatric patients and adults. Case reports or series and letters were excluded. The authors assessed the abstracts of the available literature (*n* = 171) and reviewed the full texts of potentially relevant articles (*n* = 98) that were analyzed to provide a critical discussion. In addition, the reference list of all articles was checked to identify relevant studies. The following keywords were used for the search (alone or in combination): exercise, physical activity, inflammation, obesity, pediatric obesity, obesity-related chronic inflammation, adipose tissue. PubMed, Scopus, EMBASE, and Web of Science were used (from February to April 2022) as electronic databases for this research. The contributions were collected by V.R., C.B., E.C., R.G., V.T., and V.C.P. and critically analyzed with V.C. and M.V. The resulting draft was discussed by V.C., M.V., and C.C. and critically revised by G.Z. The final version was then recirculated and approved by all.

## 3. Obesity in Pediatric Age

Obesity is defined as an excess in body fat mass. The current practical definition of obesity is generally assessed with the body mass index (BMI) and is calculated by dividing body weight in kilograms by height in meters squared. It is the standard clinical measure of overweight and obesity for children 2 years of age and older; however, BMI is a surrogate measure of adiposity, since it is reasonably reliable in the healthy pediatric population [9], but it may slightly overestimate adiposity in children who are short or have low muscle mass due to low levels of physical activity [10]. For children younger than 2 years, weight-for-length is the standard measure of overweight and obesity. Waist circumference and waist-to-hip ratio can be used to assess abdominal obesity, whereas skinfold thickness is a useful indicator of adiposity [1,11,12,13]. Due to the normal growth and development of children and adolescents, BMI cutoffs are used to report overweight and obesity in children vary with age and sex. Regarding children and adolescents between the ages of 2 and 20 years of age, overweight is referred to as the BMI being between the 85th and the 95th percentiles for age and sex; obesity is referred to as the BMI being at or above the 95th percentile for age and sex; and severe obesity is referred to as the BMI being at or above 120% of the 95th percentile [14,15]. Some experts recommend classifying obesity into three classes: class I obesity (BMI equal to or greater than the 95th percentile to less than 120% of the 95th percentile), class II (BMI equal to or greater than 120% to less than 140% of the 95th percentile, or BMI equal to or greater than 35 kg/m^2^), and class III (BMI equal to or greater than 140% of the 95th percentile, or BMI equal to or greater than 40 kg/m^2^) [16].

Although obesity rates vary among countries, in general, its prevalence has increased steadily over the past 40 years, notably doubling from 1980 to the present in more than 70 countries [17,18]. Specifically, in developed countries, its prevalence reached 23.8% for boys and 22.6% for girls in 2013 compared with 16.9% for boys and 16.2% for girls in 1980 [19]. In developing countries, prevalence also increased from 8.1% in 1980 to 12.9% in 2013 for boys and from 8.4% in 1980 to 13.4% in 2013 for girls [19]. According to a report by the WHO, 39 million children under the age of 5 showed overweight or obesity in 2020 worldwide [20]. In addition, the rate of increase in obesity is greater in pediatric age than in adulthood, and that the prevalence of childhood obesity shows the differences in the trends across countries [17,21]. Based on the Global School-Based Student Health Surveys from 2005 to 2017, the trends in overweight and obesity adolescents aged 12 to 15 years showed an increased prevalence in the Americas, Africa, and South East Asia. In contrast, the trends in the prevalence in the Eastern Mediterranean and Western Pacific plateaued, although the prevalence remained at a high level. In terms of relative change, lower-middle-income countries experienced a more rapidly increasing prevalence of overweight and obesity compared to high- and upper-middle-income countries [22].

Diet has been broadly investigated as a major cause of obesity. Rising intake of fast food and sugary beverages is closely linked to childhood obesity; over the past two decades, fast food consumption has dramatically increased in accordance with an increased prevalence of childhood obesity [21,23,24]. Obesity is assumed to be caused by increased fat intake, but it appears that besides than the amount of fat intake, the type of dietary fat (e.g., trans fat) is more significant [21,25]. Dietary patterns, such as the number, regularity, and duration of meals, are also traditionally considered as eating behaviors related to obesity to obesity [26]. For example, eating without hunger, according to an uninhibited eating pattern, leads to weight gain and binge eating in adults [21,27]. A sedentary lifestyle on its own has been identified as being a consistent risk factor for obesity and related diseases, regardless of physical activity [28,29]. Short sleep time and poor sleep quality are other risk factors for obesity [30,31]. In addition, environmental factors, such as family, school, community, and lifestyle, play an important role in the growing prevalence of obesity worldwide [32]. Since parent–child relationships influence children’s behaviors, such as their food choices and physical activity level, the home setting is definitely crucial in childhood obesity [25]. School is where children typically spend most of their time and receive their primary education; therefore, school can affect children’s behavior as well regarding food choices and physical activity [25].

Childhood and adolescence are a crucial time for shaping eating and physical activity-related behavior, and several environmental drivers influence this behavior. For instance, the restrictive measures imposed during the COVID-19 pandemic have had detrimental effects on various lifestyle components and have resulted in physical inactivity and sedentary behaviors, influencing the maintenance of weight and contributing to obesity among children and adolescents [33,34,35,36]; a decreased frequency of engaging in active transport, moderate or vigorous and high-intensity housework, physical activity (PA) during leisure time, walking during leisure time, and increased sedentary, sleeping, and screen time has been reported [37]. 

Prevention rather than treatment is the key to successful obesity control: implementing nutrition education, stimulating physical activity, and abolishing sedentary behavior are the starting points of this prevention project [21].

## 4. Adipose Tissue and Associated Inflammation

AT has been always considered as the primary site of storage for excess energy, although it is now effectively considered an endocrine organ, able to produce and release biologically active compounds involved in obesity-associated chronic inflammation and pathological metabolic processes [38].

As reported in humans and experimental models, the major cellular component of AT is represented by adipocytes, which are supported by an extracellular matrix strewn with fibroblasts, preadipocytes, endothelial cells, and immune cells [39,40]. In particular, the discovery of a strong local presence of leukocytes such as macrophages, mast cells, neutrophils, monocytes, natural killer cells (NKs), and T and B lymphocytes has led scientists to define the human AT as a real immune organ involved in delicate immune homeostasis [41,42,43]. This consideration is supported by further evidence: first, perinodal adipocytes are detectable in the AT. They almost entirely accumulate polyunsaturated fatty acids, essential for the synthesis of the main second messengers for immune cells (eicosanoids and docosanoids), and the control of their lipolytic activity is performed by lymphocytes and lymph nodal dendritic cells (DCs), confirming the close connection between AT and lymphoid structures [44]. In addition, the presence of immune structures at the level of the omental and mesenteric AT, better known as “milky spots”, assigned to immune surveillance and rich in cells with peculiar characteristics when compared to conventional secondary lymphoid organs, has been demonstrated [45,46]. Indeed, it is known that AT immune cells show distinctive traits in animals [47] and that the imbalance to which the body’s energy metabolism is subjected leads to an alteration of this profile, feeding the inflammatory response typical of obesity and contributing to the onset of related metabolic disorders in humans [48]. In conditions of positive energy balance, in fact, the AT undergoes morphological and metabolic changes that lead to the presence of acute-phase proteins and to a massive release of pro-inflammatory cytokines at the expense of those with anti-inflammatory action, thus generating signals that recall the immune cells in place and that consequently result in an increase in the inflammatory infiltrate in the AT; hence the onset of a chronic low-grade inflammation [48].

Macrophages (M) are one of the main cell populations present in AT, and depending on the expressed surface antigens and secreted molecules, they can be distinguished in ATM1 macrophages, with a typically pro-inflammatory phenotype, and ATM2 macrophages, with an anti-inflammatory phenotype [49]. In humans, while the prevailing subtype in AT is normally constituted by M2 macrophages, during obesity, there is not only an increase in terms of quantity [50] but also a shift towards the M1 subtype, which forms crown-like aggregates around the inflamed necrotic AT [51,52]. The one concerning the macrophage shift is a complex and still not fully understood process, as it seems that, in vivo, ATMs simultaneously show M1 and M2 typical markers [53,54]; however, it has been discovered that this shift could be partly induced by the recruitment of circulating monocytes, which then differentiate into M1 cells and by the stimulus to cell migration resulting from the binding interaction between monocyte chemoattractant protein-1 (MCP1), secreted by hypertrophic adipocytes, and CCR2 (C-C chemokine receptor type 2) on the macrophagic surface [55,56], although the latter event still needs further investigation. However, given the role that ATMs M2 play in maintaining adequate insulin sensitivity and glucose tolerance [57] through the secretion of anti-inflammatory cytokines such as IL-10 [51], it has been found that the shift towards M1 leads to a remarkable secretion of tumor necrosis factor (TNF)-α and interleukin (IL)6, whose elevated concentrations positively correlate with the development of insulin resistance (IR) and the onset of type 2 diabetes mellitus (DMT2) [58], thus demonstrating how macrophages are effectively involved in complications related to obesity, in both murine models and humans.

The secretion of pro-inflammatory molecules also affects mast cells and neutrophils; these cell populations are increased in conditions of obesity and, by releasing molecules such as TNF-α, IL-1b, IL-6, IL-4 and elastase, seem to promote the inflammatory state, playing a role in the onset of insulin resistance (IR) [59,60,61,62]. Among other cellular populations, CD14^++^ monocytes in adults with obesity are present in a pro-inflammatory state [63], favoring the onset of obesity-related complications such as hyperglycemia and atherosclerosis [64,65]; interestingly, this condition seems to already be present in younger patients [66].

Finally, an important role in the onset of complications related to obesity is played by B and T lymphocytes; studies on mice have shown that obesity activates B lymphocytes, which infiltrate the AT and produce antibodies capable, in turn, of activating macrophages. The action exerted by B lymphocytes promotes the progression of the inflammatory state; moreover, through the activation of T lymphocytes and the selective suppression of Treg, normally responsible for regulating the immune response itself in adults affected by obesity [67,68]. However, CD4^+^ T lymphocytes, activated by adipocytes through the expression of major histo-compatibility complex class II (MHCII), are involved in the early stages of IR [69]; in fact, it has been shown through studies on mouse models fed on a high-fat diet (HFD) that the number of CD4^+^ Th1 cells is increased compared to Th2 and Tregs cells and that this event, with consequent greater secretion of IFN-γ, correlates positively with IR [70]. A similar role is played by natural killer (NK) cells, whose presence in the AT promotes, through the secretion of IFN-γ and TNF-α, the recruitment of inflammatory T lymphocytes, macrophage infiltration, and, moreover, Th1 polarization itself [71,72,73].

Finally, it is important to underline that in subjects with obesity, there is a correlation between IL-6 secretion and the presence of a remarkable concentration of Th17 cells, T lymphocytes typically involved in inflammatory processes whose value seems to decrease after therapeutic treatments with metformin, specific diets, and exercise. Th17 lymphocytes mainly secrete IL-17, a cytokine with a pro-inflammatory action that in turn stimulates the release of various other molecules capable of activating the immune system, such as IL-6, IL-21, IL-22, TNF-α, and GM-CSF. The close relationship between obesity and Th17 lymphocytes was demonstrated in research conducted on animal models, where it was found that maternal obesity causes an inflammatory state in the intestine of the fetus, with high amounts of circulating IL-17 [74,75,76]. Treg lymphocytes, which are generally present in high concentrations and have a peculiar cytokine expression profile in the AT, appear, instead, to be decreased in conditions of obesity. Normally, they secrete large amounts of IL-10, which maintains macrophages in the alternative M2 state and performs a positive action on adipocytes by promoting insulin sensitivity; therefore, their reduction seems to promote the inflammatory state in high-fat diet-induced mice [47,51]. Interestingly, clinical research conducted on animal models fed an HFD and whose immune system was stimulated to produce high concentrations of cytokine IL10 revealed an expansion of the regulatory T subpopulation and, consequently, a significant reduction in blood glucose levels, insulin resistance, and increased glucose tolerance [77]. In both children and adults, the triggered Treg/Th17 imbalance has been the subject of recent studies and appears to be involved not only in the generation of the low-grade inflammatory state typical of obesity, but also in the onset of complications such as IR, hypertension, cardiovascular risks, and DMT2 [78,79,80]. In addition, the interconnection between the two subpopulations has been extensively evaluated in animal models, and several studies on adults have shown that, for example, IL17 cytokine is able to promote the differentiation of Th17 cells and, at the same time, inhibit Treg cells’ differentiation [81,82,83]. However, further investigation is needed in order to validate these claims. 

In addition to inflammatory immune cells, adipokines also play a critical role in obesity-related inflammation. Adipokines are adipocyte-derived molecules that regulate several pathways, including glucose and lipid metabolism. However, during chronic inflammation, that adipokine profile becomes dysregulated. Adiponectin (AdipoQ) is secreted by the healthy adipose tissue, and its levels are elevated in blood circulation in physiological conditions. In humans, AdipoQ, responsible for insulin sensitivity, increases fatty acid and suppresses hepatic glucose production. Moreover, adiponectin has a protective immunomodulatory function: in fact, it is able to reduce pro-inflammatory cytokine secretion (i.e., TNF-α, IL-6, MCP1) and to increase anti-inflammatory cytokines (such as IL-10) [84]. However, plasmatic adiponectin concentration has been observed to be reduced in patients with obesity, contributing to insulin resistance [85,86]. In addition, subjects with overweight or obesity plasma adiponectin levels are negatively correlated with waist circumference, BMI, fat mass, fat mass percent, and fat mass index [87,88]. 

It is well recognized that leptin, another hormone secreted from the adipose tissue into the bloodstream, modulates food intake by binding to its receptors in the hypothalamus [89]. However, leptin receptors have also been detected in a variety of cells types, such as in adipocytes, pancreatic beta cells, and immune cells [90]. Thus, the activation of leptin receptors in these different districts leads to a plethora of consequences. In fact, since individuals with obesity have higher plasmatic leptin levels with respect to lean subject, hyperleptinemia contributes to IR and to the low-grade inflammatory states [91,92]. Moreover, leptin promotes Th17 and pro-inflammatory cytokines, inhibiting Treg differentiation [93].

Increasing evidence has shown not only that is human resistin expressed in adipocytes and pancreatic cells, but also that it is largely produced by immune cells [94]. Because of its heterogeneous expression, it becomes clear that resistin regulates several biological processes, from insulin resistance to pro-inflammatory reactions. In fact, it has been found that resistin promotes the release of inflammatory cytokines, such as CRP, IL-1, IL-6, IL-12, and TNF-α, in both rodents and humans [95]. 

Chemerin, initially discovered as a retinoic responsive gene, has recently been identified as an adipokine [96]. It is highly expressed in the WAT as compared to the BAT, suggesting a role in both the proliferation and the differentiation of murine pre-adipocytes [97]. In addition, chemerin receptor CMKLR1 activation, being expressed by macrophages, dendritic cells, and natural killer cells, mediates their infiltration into AT [98]. Taken together, these data support the existence of a vicious crosstalk between adipocytes and immune system that promotes the maintenance of low-grade chronic inflammation in subjects with obesity.

**Figure 1:**
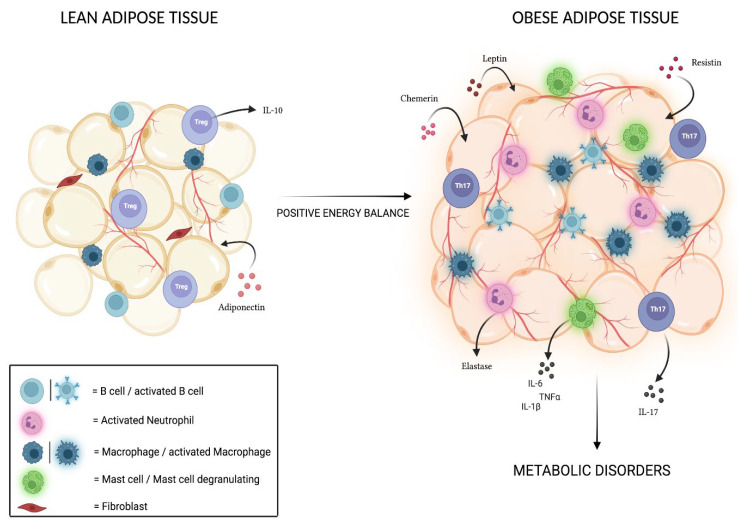
Immunomodulatory property of adipose tissue (created by using Biorender).

In Figure 1, the immunomodulatory property of adipose tissue is reassumed. 

## 5. Physical Activity Guidelines for Children and Adolescents

PA is essential for regular growth and development [99,100], has an important role in the prevention of overweight and obesity in childhood and adolescence, and reduces the health risks related to excess weight [101]. The WHO, through the Global Action Plan for Physical Activity 2018–2030 [102] and the Physical Activity Strategy for the WHO European Region 2016–2024, provided guidance and support for national policies to promote regular PA for all people. Current WHO guidelines recommend that children and adolescents between 5 and 17 years should perform at least 60 min of moderate- to vigorous-intensity daily amount of PA, which may be realized by performing activities in multiple short bouts during the day [103]. These proposals include play, games, sports, active transportation, recreation, and physical education, as well as planned exercise or training sessions [104]. It is also recommended that 30 min of daily exercise should be performed in the school routine. The structured and unstructured daily routine activities must be enjoyable and preferably sustainable [105]. 

Some children and adolescents suffer from barriers related to demographic, personal, social, and environmental circumstances that limit them from reaching their exercise and PA goals. Youngsters are greatly affected by the PA level of the people who surround them and their exercise habits. Even preschool-aged children are influenced by the amount of exercise of their parents [106]. Preschoolers who have more physically active parents exercise more than children of parents who are inactive [107]. Conversely, children who spend more time outdoors are more active than those who spend more time at home [107]. Additionally, urban habitats with limited play space and omnipresent worry about child safety contribute to spending less time outdoors, and the cost and perceived lack of access to playgrounds, supervision, and equipment contribute to employing less time outside as well [108]. Favorable predictors of PA levels include family, peer, and community stimulation; an active and sportive self-identity; the wish to avoid weight gain; personal achievement [109]; and perceived competence [110]. Children and parents together should have an active lifestyle from a very young age and not depend on the school system to maintain their children fitness level; thus, changing parental exercise habits may help with childhood fitness. Moreover, parents and schools should stimulate children and adolescents to play outside, play at recess, and walk or bike to school. Furthermore, Riddoch et al. [111] also found gender differences in PA practice across age, with boys more active than girls, especially at 9 years old (21% more active) and 15 years old (26% more active), where sex gaps in active time spent in at least moderate-intensity activities are even more pronounced (20% and 36% difference, respectively). In a study by Garaulet et al. [112], which investigated 14- to 18-year-old adolescents, overweight girls showed nearly identical levels of PA (work, sport, and recreational index) compared with their non-overweight counterparts, but overweight boys had a significantly lower sport index than their non-overweight peers. Sports clubs may be too competition-oriented and, therefore, exclude less-fit children and adolescents, especially children and adolescents with obesity. Given the commonly low levels of participation in sports by the young with obesity, the encouragement to participate in sports from an early age may be important in the prevention and treatment of weight gain.

Although regular PA practice demonstrated clear benefits and a healthy lifestyle is able to reduce cardio-metabolic risk factors in all subjects, children with obesity tend to have lower PA levels compared to their peers [105,113,114]. As reported by Yu et al. [115], children and adolescents with obesity spent 30% less of time engaging in PA pursuits and 51% more time in sedentary activities during time dedicated to waking activities. Many studies showed that children with obesity experienced difficulties to begin and maintain PA programs due to the lack of motor skills caused by excessive body weight and to the tendency to be inactive. Subsequently, children with obesity usually acquire negative feelings related to the PA practice, increasing the rate of PA abandonment and creating a vicious circle in which the sedentary habits became predominant. Moreover, Babic et al. [116] showed that adolescents with a higher level of physical self-esteem had more chances to engage in PA; conversely, lower perception in fitness could limit participation in PA/exercise programs and, consequently, the perceived enjoyment [114,117]. A previous study of Vandoni et al. [118] highlighted the importance of investigating physical self-esteem and PA pleasure prior to engage into a regular exercise program in children and adolescents. In fact, children with obesity also showed poor performance in the evaluation of motor control [118]. An accurate evaluation of self-reported physical fitness could identify low-fitness (and possibly low-activity) children in order to enact primary surveillance and design specific PA interventions to increase long-time PA adherence. 

## 6. Anti-Inflammatory Effect of Exercise in Children and Adolescents with Obesity

In the last twenty years, a large number of studies have been conducted in order to understand the influence of exercise on the inflammatory process in both the healthy and the unhealthy adult populations suffering from non-communicable diseases [5]. 

There are well-known health benefits from regular exercise, and ample evidence points to a decreased risk of developing several chronic diseases, including obesity, CVD, metabolic syndrome, T2D, and several cancers (e.g., colorectal and breast cancers), which are conditions typically associated with underlying chronic low-grade inflammation [119,120]. 

PA may successfully prevent adipose tissue accumulation through increased energy expenditure while promoting cardiovascular health by improving the blood lipid profile, which is presumed to limit the production of atherosclerosis. However, the protective effect of a physically active lifestyle against chronic disease can be further attributed to the anti-inflammatory effects of exercise [120,121]. Chronic low-grade inflammation is characterized by a two- to fourfold increase in circulating pro- and anti-inflammatory molecules compared with those observed in healthy adults [122]. 

Aadland et al., as previously stated by Ekelund et al., indicated that children and teenagers should spend time training at moderate to vigorous intensities to improve their cardio-metabolic health [123,124]. Specifically, a positive effect of PA is achieved during intermittent activities of vigorous intensity, such as those involving running and jumping. At the same time, a very weak association was found between sedentary time and metabolic health [123].

Several studies have been conducted to highlight the beneficial role of PA in chronic low-grade inflammation, and its impact on inflammation has received significant attention in recent years. 

Findings from a study performed by Haapala et al. [125] showed that higher levels of moderate physical activity (MPA) and/or vigorous physical activity (VPA) and lower levels of sedentary time (ST) were associated with lower levels of circulating biomarkers of inflammation among children. Nevertheless, the same group also showed a strong association between percentage fat mass (BF%) and circulating levels of inflammatory biomarkers. Accordingly, the link between ST, MPA, and/or VPA levels and biomarkers of inflammation in 6- to 8-year-old children also appears to be related to BF%: higher ST levels and lower PA levels were associated with higher circulating levels of inflammatory biomarkers in children with higher BF% but not in those with lower BF%. Furthermore, in relation to diet, Haapala et al. showed that PA was inversely associated with circulating levels of biomarkers of inflammation in children with a poor quality diet but not in those with a better quality diet [125].

The most common biomarker in cohort studies to reflect inflammatory status is C-reactive protein (CRP), which is widely used in clinical settings. CRP is an acute-phase protein that is a nonspecific marker of systemic inflammation; it appears to be involved in the pathogenesis of several chronic conditions, most notably CVD. A large collection of studies have consistently reported inverse associations between self-reported physical activity or objectively measured aerobic fitness with circulating levels of CRP [126]. 

In particular, a 2016 systematic review and meta-analysis of 83 randomized controlled trials suggested that exercise performed for more than 2 weeks can reduce CRP. This reduction was more evident when exercise was accompanied by a reduction in fat mass and BMI [127]. These results were confirmed by another 2019 meta-analysis, targeting the effects of aerobic exercise on CRP in healthy adults [5]. 

The mechanism through which exercise affects CRP is unclear. It could be associated with the reduction in IL-6, which induces a hepatic acute phase response and may be due to confounders such as hypertension, age, smoking, and body mass-related variables with which CRP is also associated [128]. 

Other biomarkers, particularly interleukin-6 (IL-6) and Tumor-Necrosis-Factor α (TNF-α), are increasingly reported to be inversely associated with PA [121].

Skeletal muscle, as a metabolically active organ, produces and secretes a variety of molecules in response to contraction [120,129]. The products of these tissues have been termed “myokines” and have been shown to exert many different effects, some of which occur locally within skeletal muscle itself, while others act distally in other organs and tissues including the liver, pancreas, adipose tissue, and cardiovascular system [130]. Although our understanding of skeletal muscle as an endocrine organ is currently far from complete, available evidence suggests that contraction-induced myokine production may mediate many beneficial physiological effects of regular exercise that positively impact metabolic health [131]. 

Currently, among the identified myokines, the most studied is the cytokine IL-6. During acute exercise, muscle contraction stimulates the release of IL-6 from skeletal muscle, with circulating levels increasing more than 100-fold with intense and prolonged exercise, e.g., in a marathon [120]. Generally, however, more moderate responses are observed that are proportional to exercise intensity and duration, individual fitness level (inversely), and muscle mass utilized by the specific exercise modality [132]. Skeletal muscle-derived IL-6 has been identified as a key metabolic intermediary, and research has defined IL-6 as an energy sensor, which functions to preserve fuel availability during exertion [133]. The marked increase in circulating levels of muscle skeletal-derived IL-6 is responsible for positive changes in circulating levels of several other inflammatory mediators. The levels of IL-6 secreted depend on the recruited muscle mass and the length and the intensity of exercise and is connected (inversely) to the amount of glycogen in the muscles. Subsequently, in order to promote anti-inflammatory conditions using PA, it should be of a high enough level that it uses glycogen as its primary fuel (greater than 70% of maximal aerobic capacity) and/or of sufficient length to reduce stored glycogen. Thus, a single workout is unlikely to cause adaptive changes. In contrast, the benefits produced by PA are more likely to be observed when the activity is repeated over the long term. In addition, the type of activity performed has a significant impact on the benefits obtained. Low-intensity exercise programs, such as walking and other “lifestyle”-related efforts to increase PA, for example, household tasks, are not sufficient to have a favorable impact on circulating inflammatory markers [120]. Instead, to achieve such benefits, they must be of a higher intensity, i.e., moderate-to-vigorous intensity (at least 70% of maximal aerobic capacity). A combination of aerobic activities with muscle-strengthening exercise, i.e., resistance training, will likely yield the greatest benefit, particularly regarding optimizing the anti-inflammatory effect of training.

Furthermore, in contrast to adipose-derived IL-6, which is commonly considered pro-inflammatory, episodic increases in skeletal-muscle-derived IL-6 trigger an anti-inflammatory cascade by inhibiting the release of pro-inflammatory cytokines (TNF-α and IL-1β) through stimulation of their antagonistic soluble receptors that increases and remains elevated during and after exercise [5,134,135].

Further to reducing pro-inflammatory factors, exercise-induced IL-6 also precipitates the release of IL-10, a strong anti-inflammatory molecule, which subsequently inhibits the synthesis of many pro-inflammatory mediators, such as IL-1α, IL-1β, TNF-α, the chemokines IL-8, and macrophage inflammatory protein-1α (MIP-1α) [134]. These inflammatory mediators are the main players in the distribution of inflammatory responses and the adoption of immune cells to inflammation site. Extensive exercise can also cause the release of CRP the next day [134]. This may precipitate an extra anti-inflammatory effect by triggering the induction of anti-inflammatory cytokines from circulating monocytes and reducing the synthesis of pro-inflammatory cytokines in tissue macrophages. Furthermore, exercise may also promote an acute anti-inflammatory effect by causing the production of cortisol and adrenaline, two hormones that give out potent anti-inflammatory effects and seem to be capable of reducing the production of TNF-α and IL-1β [136,137]. Muscle-derived IL-6 may in part be the cause of the cortisol reaction to exercise [120,132]. Other skeletal-muscle-derived factors that may precipitate indirect effects (by affecting adiposity) on systemic inflammation are irisin and IL-15 [129]. Irisin is a newly discovered myokine that is secreted into the circulation in high levels following exercise and has the capability to induce the transformation of white adipocytes into “brite” (brown-white) cells, which have a phenotype that resembles those of brown adipocytes (metabolically deficient cells that raise thermogenesis and energy expenditure, as opposed to white adipocytes, whose main function is lipid deposition and storage). Thus, irisin may be a mid-point with a positive contribution to energy homeostasis and metabolic regulation. IL-15 is also produced in skeletal muscle as a reaction to contraction and also seems to give favorable results on adiposity. In particular, IL-15 seems to have a beneficial effect on abdominal fat, by reducing it, as we have seen in experiments carried out on mice [120,138]. In humans, circulating IL-15 has been inversely connected to adipose tissue present at the trunk level [139]. That said, further research is required in order to determine the processes by which IL-15 influences central adiposity. Furthermore, intense exercise causes an increase in the number of circulating leukocytes and a temporary reduction in many stages of immune cell function. These effects are conditional on both the length of exercise and its intensity, with high-impact exercise (prolonged and/or high intensity) providing the most heightened effects [140]. Leukocytes release a variety of cytokines (including IL-6), which are concerned in the triggering and control of the immune response. That said, though exercise mobilizes specific groups of leukocytes that are currently expressing cytokines from marginal pools, unlike skeletal muscle, exercise does not directly contribute to cytokine release from leukocytes.

In the long term, physical exercise reduces the size of adipose tissue, which acts as an endocrine and paracrine organ responsible for stimulating the increase in inflammatory mediators. In support of this fact, some studies demonstrated that there is an overexpression of IL-6 and TNF-α deriving from macrophages residing in adipose tissue in individuals with obesity and overweight compared with normal weight adults [5]. 

Leptin and adiponectin are two adipokines that are cytokines produced by adipose tissue and that act on several target organs, including brain, liver, pancreas, muscle, immune system, and adipose tissue itself. Apart from their metabolic effect, both are involved in inflammation and immune response, with leptin having inflammatory and adiponectin having anti-inflammatory properties. A high level of leptin activates monocytes’ and macrophages’ production of IL-6 and TNF-α. The latter stimulates the expression of leptin and its receptor. On the other hand, adiponectin reduces TNF-α production in macrophages. The levels of leptin and adiponectin correlate with BMI, positively and negatively, respectively; in addition, for these reasons, adipose tissue reduction is associated with inflammation decrease [141]. 

Tenòrio et al. [142] performed a 6-month randomized exercise intervention study in adolescents to evaluate how biomarkers of inflammation and endothelial dysfunction change according to two different training intensities. 

It has been observed that both low-intensity exercise (low intensity training (LIT) and high-intensity exercise (HIT) are able to significantly reduce the concentration of leptin; however, there was a reduction in circulating levels of neutrophils, monocytes, IL6, and TNF-α. Therefore, it is apparent that there is a potential superiority of aerobic training performed at high-intensity over lower-intensity training in successfully modifying specific biomarkers of CVD.

Yet another study carried out in children by Merlin et al. [143] comparing serum adiponectin concentration and metabolic health level took the mean of daily steps as an index of the intensity of physical activity. Children were clustered into two groups: cluster 1 performed between 7039 and 11,023 daily steps (sedentary group), while cluster 2 showed daily steps limits between 12,009 and 15,739 (active group). 

This suggests that less physically active children present a predominance of Th1 response for lymphocyte differentiation, with a higher production of IFN-gamma and TNF-alpha, lower adiponectin, and higher leptin plasmatic concentration. 

On the other hand, more physically active children present an anti-inflammatory response, especially of regulatory T cells. To conclude, children are represented by two different levels of PA based on the average of the daily steps, and these differences are reflected by their body composition and inflammatory responses. The sedentary group of children, characterized by lower PA level, displayed higher BMI, body fat, and increased Th1 cells, which h can be modulated by the higher production of leptin. The active group, characterized by higher levels of PA, had lower BMI and body fat, as well as higher Treg cell differentiation.

Furthermore, during exercise, there is a rapid elevation of PGC1-α via AMPK followed by a return to baseline level post-exercise. PCG1-α is a transcriptional co-activator that regulates genes involved in energy metabolism and regulates mitochondrial biogenesis. Increased expression of PCG1-α is associated with changes in the musculature related to long-term exercise, such as fiber-type switching toward the more oxidative types I and IIa. This co-activator is also associated with the polarization from M1 pro-inflammatory to M2 anti-inflammatory macrophages and the downregulation of oxidative stress-mediating gene expression in vascular endothelial cells [5]. 

Chronic exercise has been demonstrated to decrease cell-surface expression of TLR receptor on immune cells [144]. On the other hand, physical inactivity is associated with an augmented TRL activation. TLRs are transmembrane proteins that play an important role in the recognition of microbial pathogens and endogenous danger signals of tissue damage and their activation causes systemic inflammation and is associated with chronic non-communicable diseases [145]. 

PA can directly regulate the immune system and has the potential to indirectly affect chronic disease by modulating our immunological response [7,146,147,148,149,150]. The earliest studies regarding exercise’s effect on immune systems began in the mid-1980s and were conducted by David Nieman. He observed that exercising regularly with moderate intensity is beneficial for our bodies, whereas sustained bouts of intensive training can depress immunity [151]. Both acute and chronic exercise have shown a remarkable response in leukocyte redistribution, activity, trafficking, and function [140,152]. Intensity, duration, and volume of PA have all been reported to influence the exercise-associated redistribution of immune cells in the circulation in all people [146,153,154]. Our immune system is composed of two main effectors: the innate immune system and the adaptive immune system. The innate, or nonspecific, immune system includes both cells (neutrophils, macrophages, dendritic cells (DCs) and natural killer (NK) cells) and soluble factors (complement proteins and antimicrobial peptides) and is the first line of defense against pathogens. These cells appear to increase immediately after exercise and then return to pre-exercise levels within 6 to 24 h after cessation of exercise [146,155,156,157]. On the other hand, adaptive immunity, also known as acquired or specific immunity, is composed of T and B lymphocytes and can create an immunological memory. T cells play an important role in cell-mediated immune responses, whereas B cells are intimately involved in the humoral immune response [158]. It is widely accepted that, proportional to the duration and intensity of exercise, lymphocytosis occurs during and immediately after exercise, falling below pre-exercise levels during the early stages of recovery [146,148,159]. Regarding the effect of exercise on the humoral component of the immune response, studies have reported that serum Ig concentration appears to remain slightly increased or unchanged [134,146].

Evidence exists for an inverse association between exercise and inflammatory factors in adults, yet evidence of these relationships among youth is inconsistent [160,161]. Some studies support an inverse association between exercise and CRP, IL-6, and fibrinogen (Fg) in youth, but there are also other studies that state the opposite [160]. Some observational studies conducted on children and adolescents have shown that a low-grade inflammatory state is associated with adiposity in children and adolescents, as has been demonstrated in adults [162,163,164]. Low-grade inflammation is associated with anthropometric measures such as body mass index (BMI) and waist circumference in adolescents, but data on children are sparse [162,163]. A school-based cross-sectional study of children aged 9.5 +/− 0.4 years, involving 233 Swedish children and conducted by Ruiz et al., showed that levels of low-grade inflammatory markers (CRP and C3) were negatively associated with cardiovascular fitness (CVF) and positively associated with body fat in prepubertal children [165]. There is a limitation due to the one-dimensional nature of CVF and body fat, which makes it difficult to distinguish their separate influences in an observational study. Examining the influence of the individual variables, however, it was observed that each explained a significant portion of the variance in most variables, which are CRP, Fg, C3, and IL-6, but considering them all together, only body fat maintained a significant role [165]. It was shown that the change in CVF was significantly explained by at least moderate-vigorous PA in children and adolescents, practiced on a regular basis [165,166,167]. Therefore, this confirms that PA has a positive impact on low-grade inflammation through improvement of CVF. Indeed, there are no studies examining the association of low-grade inflammation with objectively measured PA in prepubertal children. However, it is noteworthy that in this study by Ruiz et al., inflammatory markers were not associated with measures of central adiposity (waist circumference, waist-to-hip ratio, and waist-to-height ratio). A limitation of this study is the cross-sectional design, which precludes definitive conclusions about whether changes in PA, CVF, and fat cause changes in low-grade inflammation in prepubertal children. Results showed that levels of low-grade inflammatory markers were negatively associated with CVF and positively associated with body fat in prepubertal children. For most variables, the influence of fat was slightly greater than that of CVF. The results suggest that the potential beneficial effects of PA on low-grade inflammation may be explained by its association with CVF.

Platat et al. [168] demonstrated that IL-6 and insulin resistance were negatively associated with organized leisure-time PA, independent of adiposity and fat disposition, in 12-year-old children. Similarly, Cook et al. [169] found a borderline significant negative association of CRP levels with self-reported PA after adjusting for weight index in 10- to 11-year-old children. However, in both studies, a limiting element was the assessment of PA by questionnaires, which may introduce imprecision, especially in children [170].

Additionally, studies involving younger people often tend to use a single baseline measurement of CRP, IL-6, or Fg, and it is possible that this does not indicate accurately chronic inflammatory status. PA is particularly challenging to assess in young people [171]. Self-reporting is often the tool of choice, especially in epidemiological studies conducted on a large population scale. However, accuracy of recall among children and adolescents is variable, as they practice frequent but short periods of activity. To date, a limited number of interventional studies have been conducted to evaluate regular PA impact of on inflammatory factors in young people. Nevertheless, there is some evidence suggesting that a beneficial association could be observed as early as childhood or adolescence. Yet, it remains unknown how factors such as duration, intensity, and mode of exercise may influence CRP, IL-6, and Fg levels in youth. Even though several studies have demonstrated that PA is not related to inflammatory status, is it possible that blood concentrations in healthy young people, including those who may present overweight or obesity, are still not sufficiently high that regular exercise will have any appreciable effect. 

A meta-analysis by Han et al. [172] highlighted distinct mechanisms that may be responsible for the reduced levels of proinflammatory cytokines after exercise in children and teenagers. Firstly, PA could improve inflammatory cytokines by lowering the visceral fat mass; in fact there is a statistically significant positive relationship between the CRP level and a change in BMI. In addition, PA may change macrophage activation, producing a phenotypic switch from the M1 macrophage (pro-inflammatory) to the M2 macrophage (anti-inflammatory). Therefore, this meta-analysis by Han et al. [172] agrees with the theory that PA could be used as a therapy for children and adolescents in order to reverse the low-grade inflammatory state of overweight/obesity, resulting in a restoration of anti-inflammatory levels the same as those seen in normal-weight children and adolescents.

**Figure 2:**
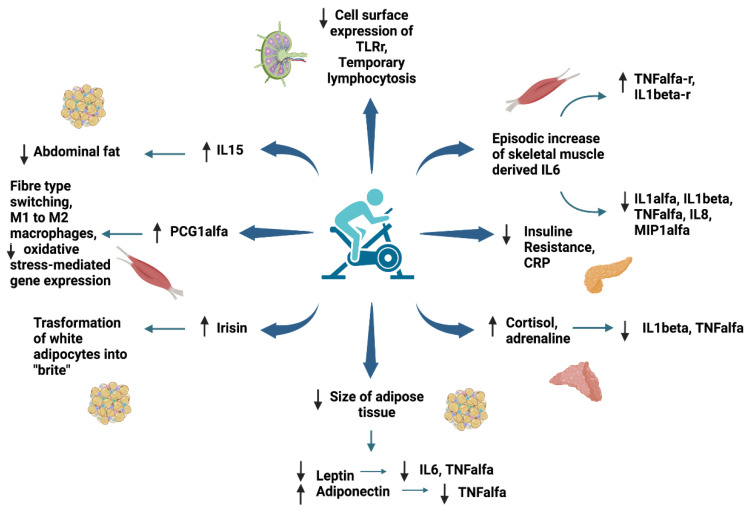
Anti-inflammatory effect of exercise in children and adolescents with obesity (created using Biorender). TNF = tumor necrosis factor; IL = interleukin; M = macrophages; TLR = Toll-like receptor; CRP = C-reactive protein; MIP = macrophage inflammatory protein.

In Figure 2, the anti-inflammatory effects of exercise are presented.

## 7. Conclusions

The prevalence of obesity in children and adolescents has increased worldwide and represents a major public health challenge. The main prevention and treatment of pediatric obesity remains lifestyle modifications, including eating habits and training. In fact, lack of physical activity and increased sedentary time are strongly associated with obesity. PE can mitigate the global problems of obesity and related comorbidities, thereby improving cardiovascular fitness and impacting metabolic profile. Moreover, the exercise-linked improvement in immunological response and the attenuation of low-grade chronic inflammation, whose hallmark is represented by cytokine release, provide benefits at systemic level. The identification of skeletal muscle as an endocrine organ, producing a wide range of metabolic factors, such as myokines, has provided a mechanistic link between muscle contraction and its beneficial influence on systemic inflammation and health. PA represents a non-pharmacological treatment to reverse the low-grade inflammatory state of overweight/obesity.

Regular PE, associated with nutrition intervention, must be considered as a natural part of a healthy lifestyle and promotes physical activity starting from childhood, and it is useful to limit the negative effects of obesity on health. 

## Data Availability

Not applicable.

## References

[1] Kumar S., Kelly A.S. (2017). Review of Childhood Obesity: From Epidemiology, Etiology, and Comorbidities to Clinical Assessment and Treatment. Mayo Clin. Proc..

[2] Ogden C.L., Carroll M.D., Kit B.K., Flegal K.M. (2014). Prevalence of Childhood and Adult Obesity in the United States, 2011-2012. JAMA.

[3] Dietz W.H., Robinson T.N. (2005). Overweight Children and Adolescents. N. Engl. J. Med..

[4] Juonala M., Magnussen C.G., Berenson G.S., Venn A., Burns T.L., Sabin M.A., Srinivasan S.R., Daniels S.R., Davis P.H., Chen W. (2011). Childhood Adiposity, Adult Adiposity, and Cardiovascular Risk Factors. N. Engl. J. Med..

[5] Metsios G.S., Moe R.H., Kitas G.D. (2020). Exercise and Inflammation. Best Pract. Res. Clin. Rheumatol..

[6] Calcaterra V., Zuccotti G. (2022). Physical Exercise as a Non-Pharmacological Intervention for Attenuating Obesity-Related Complications in Children and Adolescents. Int. J. Environ. Res. Public Health.

[7] Gleeson M., Bishop N.C., Stensel D.J., Lindley M.R., Mastana S.S., Nimmo M.A. (2011). The Anti-Inflammatory Effects of Exercise: Mechanisms and Implications for the Prevention and Treatment of Disease. Nat. Rev. Immunol..

[8] Gregory A.T., Denniss A.R. (2018). An Introduction to Writing Narrative and Systematic Reviews—Tasks, Tips and Traps for Aspiring Authors. Heart Lung Circ..

[9] Freedman D.S., Sherry B. (2009). The Validity of BMI as an Indicator of Body Fatness and Risk Among Children. Pediatrics.

[10] Javed A., Jumean M., Murad M.H., Okorodudu D., Kumar S., Somers V.K., Sochor O., Lopez-Jimenez F. (2015). Diagnostic Performance of Body Mass Index to Identify Obesity as Defined by Body Adiposity in Children and Adolescents: A Systematic Review and Meta-Analysis: Diagnostic Performance of BMI to Identify Obesity. Pediatric Obes..

[11] Lee S., Bacha F., Gungor N., Arslanian S.A. (2006). Waist Circumference Is an Independent Predictor of Insulin Resistance in Black and White Youths. J. Pediatrics.

[12] Fernández J.R., Redden D.T., Pietrobelli A., Allison D.B. (2004). Waist Circumference Percentiles in Nationally Representative Samples of African-American, European-American, and Mexican-American Children and Adolescents. J. Pediatrics.

[13] Moreno L., Rodríguez G., Guillén J., Rabanaque M., León J., Ariño A. (2002). Anthropometric Measurements in Both Sides of the Body in the Assessment of Nutritional Status in Prepubertal Children. Eur. J. Clin. Nutr..

[14] Flegal K.M., Wei R., Ogden C.L., Freedman D.S., Johnson C.L., Curtin L.R. (2009). Characterizing Extreme Values of Body Mass Index–for-Age by Using the 2000 Centers for Disease Control and Prevention Growth Charts. Am. J. Clin. Nutr..

[15] Gulati A.K., Kaplan D.W., Daniels S.R. (2012). Clinical Tracking of Severely Obese Children: A New Growth Chart. Pediatrics.

[16] Skinner A.C., Skelton J.A. (2014). Prevalence and Trends in Obesity and Severe Obesity Among Children in the United States, 1999-2012. JAMA Pediatr..

[17] (2017). The GBD 2015 Obesity Collaborators Health Effects of Overweight and Obesity in 195 Countries over 25 Years. N. Engl. J. Med..

[18] Gregg E.W., Shaw J.E. (2017). Global Health Effects of Overweight and Obesity. N. Engl. J. Med..

[19] Ng M., Fleming T., Robinson M., Thomson B., Graetz N., Margono C., Mullany E.C., Biryukov S., Abbafati C., Abera S.F. (2014). Global, Regional, and National Prevalence of Overweight and Obesity in Children and Adults during 1980–2013: A Systematic Analysis for the Global Burden of Disease Study 2013. Lancet.

[20] WHO Obesity and Overweight. https://www.who.int/news-room/fact-sheets/detail/obesity-and-overweight.

[21] Lee E.Y., Yoon K.-H. (2018). Epidemic Obesity in Children and Adolescents: Risk Factors and Prevention. Front. Med..

[22] Fan H., Zhang X. (2022). Recent Trends in Overweight and Obesity in Adolescents Aged 12 to 15 Years across 21 Countries. Pediatric Obes..

[23] Nielsen S. (2002). Trends in Food Locations and Sources among Adolescents and Young Adults. Prev. Med..

[24] Paeratakul S., Ferdinand D.P., Champagne C.M., Ryan D.H., Bray G.A. (2003). Fast-Food Consumption among US Adults and Children: Dietary and Nutrient Intake Profile. J. Am. Diet. Assoc..

[25] Ebbeling C.B., Pawlak D.B., Ludwig D.S. (2002). Childhood Obesity: Public-Health Crisis, Common Sense Cure. Lancet.

[26] Miller J.L., Couch J., Schwenk K., Long M., Towler S., Theriaque D.W., He G., Liu Y., Driscoll D.J., Leonard C.M. (2009). Early Childhood Obesity Is Associated With Compromised Cerebellar Development. Dev. Neuropsychol..

[27] Fisher J.O., Birch L.L. (2002). Eating in the Absence of Hunger and Overweight in Girls from 5 to 7 y of Age. Am. J. Clin. Nutr..

[28] Arluk S.L., Branch J.D., Swain D.P., Dowling E.A. (2003). Childhood Obesity’s Relationship to Time Spent in Sedentary Behavior. Mil. Med..

[29] Vicente-Rodríguez G., Rey-López J.P., Martín-Matillas M., Moreno L.A., Wärnberg J., Redondo C., Tercedor P., Delgado M., Marcos A., Castillo M. (2008). Television Watching, Videogames, and Excess of Body Fat in Spanish Adolescents: The AVENA Study. Nutrition.

[30] Jiang F., Zhu S., Yan C., Jin X., Bandla H., Shen X. (2009). Sleep and Obesity in Preschool Children. J. Pediatrics.

[31] Reilly J.J., Armstrong J., Dorosty A.R., Emmett P.M., Ness A., Rogers I., Steer C., Sherriff A. (2005). Early Life Risk Factors for Obesity in Childhood: Cohort Study. BMJ.

[32] Karnik S., Kanekar A. (2012). Childhood Obesity: A Global Public Health Crisis. Int. J. Prev. Med..

[33] Gontariuk M., Krafft T., Rehbock C., Townend D., Van der Auwermeulen L., Pilot E. (2021). The European Union and Public Health Emergencies: Expert Opinions on the Management of the First Wave of the COVID-19 Pandemic and Suggestions for Future Emergencies. Front. Public Health.

[34] Tornaghi M., Lovecchio N., Vandoni M., Chirico A., Codella R. (2021). Physical Activity Levels across COVID-19 Outbreak in Youngsters of Northwestern Lombardy. J. Sports Med. Phys. Fit..

[35] Di Renzo L., Gualtieri P., Pivari F., Soldati L., Attinà A., Cinelli G., Leggeri C., Caparello G., Barrea L., Scerbo F. (2020). Eating Habits and Lifestyle Changes during COVID-19 Lockdown: An Italian Survey. J. Transl. Med..

[36] Calcaterra V., Verduci E., Vandoni M., Rossi V., Di Profio E., Carnevale Pellino V., Tranfaglia V., Pascuzzi M.C., Borsani B., Bosetti A. (2021). Telehealth: A Useful Tool for the Management of Nutrition and Exercise Programs in Pediatric Obesity in the COVID-19 Era. Nutrients.

[37] Vancini R.L., Andrade M.S., Viana R.B., Nikolaidis P.T., Knechtle B., Campanharo C.R.V., de Almeida A.A., Gentil P., de Lira C.A.B. (2021). Physical Exercise and COVID-19 Pandemic in PubMed: Two Months of Dynamics and One Year of Original Scientific Production. Sports Med. Health Sci..

[38] Coelho M., Oliveira T., Fernandes R. (2013). State of the Art Paper Biochemistry of Adipose Tissue: An Endocrine Organ. Arch. Med. Sci..

[39] Qi Y., Hui X. (2022). The Shades of Grey in Adipose Tissue Reprogramming. Biosci. Rep..

[40] Curat C.A., Miranville A., Sengenès C., Diehl M., Tonus C., Busse R., Bouloumié A. (2004). From Blood Monocytes to Adipose Tissue-Resident Macrophages: Induction of Diapedesis by Human Mature Adipocytes. Diabetes.

[41] Frayn K.N., Karpe F., Fielding B.A., Macdonald I.A., Coppack S.W. (2003). Integrative Physiology of Human Adipose Tissue. Int. J. Obes. Relat. Metab. Disord.

[42] Lynch L., O’Shea D., Winter D.C., Geoghegan J., Doherty D.G., O’Farrelly C. (2009). Invariant NKT Cells and CD1d(+) Cells Amass in Human Omentum and Are Depleted in Patients with Cancer and Obesity. Eur. J. Immunol..

[43] Wu D., Molofsky A.B., Liang H.-E., Ricardo-Gonzalez R.R., Jouihan H.A., Bando J.K., Chawla A., Locksley R.M. (2011). Eosinophils Sustain Adipose Alternatively Activated Macrophages Associated with Glucose Homeostasis. Science.

[44] Pond C.M. (2005). Adipose Tissue and the Immune System. Prostaglandins Leukot. Essent. Fat. Acids.

[45] Kaminski D.A., Randall T.D. (2010). Adaptive Immunity and Adipose Tissue Biology. Trends Immunol..

[46] Moro K., Yamada T., Tanabe M., Takeuchi T., Ikawa T., Kawamoto H., Furusawa J.-I., Ohtani M., Fujii H., Koyasu S. (2010). Innate Production of T(H)2 Cytokines by Adipose Tissue-Associated c-Kit(+)Sca-1(+) Lymphoid Cells. Nature.

[47] Feuerer M., Herrero L., Cipolletta D., Naaz A., Wong J., Nayer A., Lee J., Goldfine A.B., Benoist C., Shoelson S. (2009). Lean, but Not Obese, Fat Is Enriched for a Unique Population of Regulatory T Cells That Affect Metabolic Parameters. Nat. Med..

[48] Bambace C., Pedrotti M., Ferrara G., Zamboni M. (2011). Obesità, Tessuto Adiposo e Infiammazione. Biochim. Clin..

[49] Gordon S. (2003). Alternative Activation of Macrophages. Nat. Rev. Immunol..

[50] Harman-Boehm I., Blüher M., Redel H., Sion-Vardy N., Ovadia S., Avinoach E., Shai I., Klöting N., Stumvoll M., Bashan N. (2007). Macrophage Infiltration into Omental versus Subcutaneous Fat across Different Populations: Effect of Regional Adiposity and the Comorbidities of Obesity. J. Clin. Endocrinol. Metab..

[51] Lumeng C.N., Bodzin J.L., Saltiel A.R. (2007). Obesity Induces a Phenotypic Switch in Adipose Tissue Macrophage Polarization. J. Clin. Invest..

[52] Wentworth J.M., Naselli G., Brown W.A., Doyle L., Phipson B., Smyth G.K., Wabitsch M., O’Brien P.E., Harrison L.C. (2010). Pro-Inflammatory CD11c+CD206+ Adipose Tissue Macrophages Are Associated with Insulin Resistance in Human Obesity. Diabetes.

[53] Bourlier V., Zakaroff-Girard A., Miranville A., De Barros S., Maumus M., Sengenes C., Galitzky J., Lafontan M., Karpe F., Frayn K.N. (2008). Remodeling Phenotype of Human Subcutaneous Adipose Tissue Macrophages. Circulation.

[54] Shaul M.E., Bennett G., Strissel K.J., Greenberg A.S., Obin M.S. (2010). Dynamic, M2-like Remodeling Phenotypes of CD11c+ Adipose Tissue Macrophages during High-Fat Diet--Induced Obesity in Mice. Diabetes.

[55] Christiansen T., Richelsen B., Bruun J.M. (2005). Monocyte Chemoattractant Protein-1 Is Produced in Isolated Adipocytes, Associated with Adiposity and Reduced after Weight Loss in Morbid Obese Subjects. Int. J. Obes..

[56] Gerhardt C.C., Romero I.A., Cancello R., Camoin L., Strosberg A.D. (2001). Chemokines Control Fat Accumulation and Leptin Secretion by Cultured Human Adipocytes. Mol. Cell. Endocrinol..

[57] Kang Y.-H., Cho M.-H., Kim J.-Y., Kwon M.-S., Peak J.-J., Kang S.-W., Yoon S.-Y., Song Y. (2016). Impaired Macrophage Autophagy Induces Systemic Insulin Resistance in Obesity. Oncotarget.

[58] Weisberg S.P., McCann D., Desai M., Rosenbaum M., Leibel R.L., Ferrante A.W. (2003). Obesity Is Associated with Macrophage Accumulation in Adipose Tissue. J. Clin. Invest..

[59] Abraham S.N., St John A.L. (2010). Mast Cell-Orchestrated Immunity to Pathogens. Nat. Rev. Immunol..

[60] Liu J., Divoux A., Sun J., Zhang J., Clément K., Glickman J.N., Sukhova G.K., Wolters P.J., Du J., Gorgun C.Z. (2009). Genetic Deficiency and Pharmacological Stabilization of Mast Cells Reduce Diet-Induced Obesity and Diabetes in Mice. Nat. Med..

[61] Nijhuis J., Rensen S.S., Slaats Y., van Dielen F.M.H., Buurman W.A., Greve J.W.M. (2009). Neutrophil Activation in Morbid Obesity, Chronic Activation of Acute Inflammation. Obesity.

[62] Talukdar S., Oh D.Y., Bandyopadhyay G., Li D., Xu J., McNelis J., Lu M., Li P., Yan Q., Zhu Y. (2012). Neutrophils Mediate Insulin Resistance in Mice Fed a High-Fat Diet through Secreted Elastase. Nat. Med..

[63] Kullo I.J., Hensrud D.D., Allison T.G. (2002). Comparison of Numbers of Circulating Blood Monocytes in Men Grouped by Body Mass Index (<25, 25 to <30, > or =30). Am. J. Cardiol..

[64] Poitou C., Dalmas E., Renovato M., Benhamo V., Hajduch F., Abdennour M., Kahn J.-F., Veyrie N., Rizkalla S., Fridman W.-H. (2011). CD14dimCD16+ and CD14+CD16+ Monocytes in Obesity and during Weight Loss: Relationships with Fat Mass and Subclinical Atherosclerosis. Arter. Thromb. Vasc. Biol.

[65] Satoh N., Shimatsu A., Himeno A., Sasaki Y., Yamakage H., Yamada K., Suganami T., Ogawa Y. (2010). Unbalanced M1/M2 Phenotype of Peripheral Blood Monocytes in Obese Diabetic Patients: Effect of Pioglitazone. Diabetes Care.

[66] Schipper H.S., Nuboer R., Prop S., van den Ham H.J., de Boer F.K., Kesmir Ç., Mombers I.M.H., van Bekkum K.A., Woudstra J., Kieft J.H. (2012). Systemic Inflammation in Childhood Obesity: Circulating Inflammatory Mediators and Activated CD14++ Monocytes. Diabetologia.

[67] DeFuria J., Belkina A.C., Jagannathan-Bogdan M., Snyder-Cappione J., Carr J.D., Nersesova Y.R., Markham D., Strissel K.J., Watkins A.A., Zhu M. (2013). B Cells Promote Inflammation in Obesity and Type 2 Diabetes through Regulation of T-Cell Function and an Inflammatory Cytokine Profile. Proc. Natl. Acad. Sci. USA.

[68] Winer D.A., Winer S., Shen L., Wadia P.P., Yantha J., Paltser G., Tsui H., Wu P., Davidson M.G., Alonso M.N. (2011). B Cells Promote Insulin Resistance through Modulation of T Cells and Production of Pathogenic IgG Antibodies. Nat. Med..

[69] Kintscher U., Hartge M., Hess K., Foryst-Ludwig A., Clemenz M., Wabitsch M., Fischer-Posovszky P., Barth T.F.E., Dragun D., Skurk T. (2008). T-Lymphocyte Infiltration in Visceral Adipose Tissue: A Primary Event in Adipose Tissue Inflammation and the Development of Obesity-Mediated Insulin Resistance. Arterioscler. Thromb. Vasc. Biol..

[70] Stolarczyk E., Vong C.T., Perucha E., Jackson I., Cawthorne M.A., Wargent E.T., Powell N., Canavan J.B., Lord G.M., Howard J.K. (2013). Improved Insulin Sensitivity despite Increased Visceral Adiposity in Mice Deficient for the Immune Cell Transcription Factor T-Bet. Cell Metab..

[71] Bellora F., Castriconi R., Dondero A., Reggiardo G., Moretta L., Mantovani A., Moretta A., Bottino C. (2010). The Interaction of Human Natural Killer Cells with Either Unpolarized or Polarized Macrophages Results in Different Functional Outcomes. Proc. Natl. Acad. Sci. USA.

[72] Martín-Fontecha A., Thomsen L.L., Brett S., Gerard C., Lipp M., Lanzavecchia A., Sallusto F. (2004). Induced Recruitment of NK Cells to Lymph Nodes Provides IFN-Gamma for T(H)1 Priming. Nat. Immunol..

[73] Martinez F.O., Helming L., Gordon S. (2009). Alternative Activation of Macrophages: An Immunologic Functional Perspective. Annu. Rev. Immunol..

[74] Ahmed M., Gaffen S.L. (2010). IL-17 in Obesity and Adipogenesis. Cytokine Growth Factor Rev..

[75] Endo Y., Yokote K., Nakayama T. (2017). The Obesity-Related Pathology and Th17 Cells. Cell Mol. Life Sci..

[76] Touch S., Clément K., André S. (2017). T Cell Populations and Functions Are Altered in Human Obesity and Type 2 Diabetes. Curr. Diab. Rep..

[77] Wang M., Chen F., Wang J., Zeng Z., Yang Q., Shao S. (2018). Th17 and Treg Lymphocytes in Obesity and Type 2 Diabetic Patients. Clin. Immunol..

[78] Calcaterra V., Croce S., Vinci F., De Silvestri A., Cordaro E., Regalbuto C., Zuccotti G.V., Mameli C., Albertini R., Avanzini M.A. (2020). Th17 and Treg Balance in Children With Obesity and Metabolically Altered Status. Front. Pediatr..

[79] Martinez-Sanchez M.E., Hiriart M., Alvarez-Buylla E.R. (2017). The CD4+ T Cell Regulatory Network Mediates Inflammatory Responses during Acute Hyperinsulinemia: A Simulation Study. BMC Syst. Biol..

[80] Zeng C., Shi X., Zhang B., Liu H., Zhang L., Ding W., Zhao Y. (2012). The Imbalance of Th17/Th1/Tregs in Patients with Type 2 Diabetes: Relationship with Metabolic Factors and Complications. J. Mol. Med..

[81] Durant L., Watford W.T., Ramos H.L., Laurence A., Vahedi G., Wei L., Takahashi H., Sun H.-W., Kanno Y., Powrie F. (2010). Diverse Targets of the Transcription Factor STAT3 Contribute to T Cell Pathogenicity and Homeostasis. Immunity.

[82] Laurence A., Amarnath S., Mariotti J., Kim Y.C., Foley J., Eckhaus M., O’Shea J.J., Fowler D.H. (2012). STAT3 Transcription Factor Promotes Instability of NTreg Cells and Limits Generation of ITreg Cells during Acute Murine Graft-versus-Host Disease. Immunity.

[83] Laurence A., Tato C.M., Davidson T.S., Kanno Y., Chen Z., Yao Z., Blank R.B., Meylan F., Siegel R., Hennighausen L. (2007). Interleukin-2 Signaling via STAT5 Constrains T Helper 17 Cell Generation. Immunity.

[84] Choi H.M., Doss H.M., Kim K.S. (2020). Multifaceted Physiological Roles of Adiponectin in Inflammation and Diseases. Int. J. Mol. Sci..

[85] Liu C., Feng X., Li Q., Wang Y., Li Q., Hua M. (2016). Adiponectin, TNF-α and Inflammatory Cytokines and Risk of Type 2 Diabetes: A Systematic Review and Meta-Analysis. Cytokine.

[86] Hotamisligil G.S. (2017). Foundations of Immunometabolism and Implications for Metabolic Health and Disease. Immunity.

[87] Gariballa S., Alkaabi J., Yasin J., Al Essa A. (2019). Total Adiponectin in Overweight and Obese Subjects and Its Response to Visceral Fat Loss. BMC Endocr. Disord..

[88] Bellissimo M.P., Hsu E., Hao L., Easley K., Martin G.S., Ziegler T.R., Alvarez J.A. (2021). Relationships between Plasma Apelin and Adiponectin with Normal Weight Obesity, Body Composition, and Cardiorespiratory Fitness in Working Adults. J. Clin. Transl. Endocrinol..

[89] Obradovic M., Sudar-Milovanovic E., Soskic S., Essack M., Arya S., Stewart A.J., Gojobori T., Isenovic E.R. (2021). Leptin and Obesity: Role and Clinical Implication. Front. Endocrinol..

[90] Holt E.H., Lupsa B., Lee G.S., Bassyouni H., Peery H.E., Goodman H.M. (2021). Goodman’s Basic Medical Endocrinology.

[91] Balland E., Chen W., Tiganis T., Cowley M.A. (2019). Persistent Leptin Signaling in the Arcuate Nucleus Impairs Hypothalamic Insulin Signaling and Glucose Homeostasis in Obese Mice. Neuroendocrinology.

[92] Abella V., Scotece M., Conde J., Pino J., Gonzalez-Gay M.A., Gómez-Reino J.J., Mera A., Lago F., Gómez R., Gualillo O. (2017). Leptin in the Interplay of Inflammation, Metabolism and Immune System Disorders. Nat. Rev. Rheumatol..

[93] Moraes-Vieira P.M.M., Larocca R.A., Bassi E.J., Peron J.P.S., Andrade-Oliveira V., Wasinski F., Araujo R., Thornley T., Quintana F.J., Basso A.S. (2014). Leptin Deficiency Impairs Maturation of Dendritic Cells and Enhances Induction of Regulatory T and Th17 Cells: Immunomodulation. Eur. J. Immunol..

[94] Su K., Li Y., Zhang D., Yuan J., Zhang C., Liu Y., Song L., Lin Q., Li M., Dong J. (2019). Relation of Circulating Resistin to Insulin Resistance in Type 2 Diabetes and Obesity: A Systematic Review and Meta-Analysis. Front. Physiol..

[95] Tripathi D., Kant S., Pandey S., Ehtesham N.Z. (2020). Resistin in Metabolism, Inflammation, and Disease. FEBS J..

[96] Karczewska-Kupczewska M., Nikołajuk A., Stefanowicz M., Matulewicz N., Kowalska I., Strączkowski M. (2020). Serum and Adipose Tissue Chemerin Is Differentially Related to Insulin Sensitivity. Endocr. Connect..

[97] Jiang Y., Liu P., Jiao W., Meng J., Feng J. (2018). Gax Suppresses Chemerin/CMKLR1-induced Preadipocyte Biofunctions through the Inhibition of Akt/MTOR and ERK Signaling Pathways. J. Cell. Physiol..

[98] Helfer G., Wu Q.-F. (2018). Chemerin: A Multifaceted Adipokine Involved in Metabolic Disorders. J. Endocrinol..

[99] Hills A.P., King N.A., Armstrong T.P. (2007). The Contribution of Physical Activity and Sedentary Behaviours to the Growth and Development of Children and Adolescents: Implications for Overweight and Obesity. Sports Med..

[100] Hills A.P., Okely A.D., Baur L.A. (2010). Addressing Childhood Obesity through Increased Physical Activity. Nat. Rev. Endocrinol..

[101] Strong W.B., Malina R.M., Blimkie C.J.R., Daniels S.R., Dishman R.K., Gutin B., Hergenroeder A.C., Must A., Nixon P.A., Pivarnik J.M. (2005). Evidence Based Physical Activity for School-Age Youth. J. Pediatrics.

[102] World Health Organization (2019). Global Action Plan on Physical Activity 2018–2030: More Active People for a Healthier World.

[103] Bull F.C., Al-Ansari S.S., Biddle S., Borodulin K., Buman M.P., Cardon G., Carty C., Chaput J.-P., Chastin S., Chou R. (2020). World Health Organization 2020 Guidelines on Physical Activity and Sedentary Behaviour. Br. J. Sports Med..

[104] McMurray R.G., Berry D.C., Schwartz T.A., Hall E.G., Neal M.N., Li S., Lam D. (2015). Relationships of Physical Activity and Sedentary Time in Obese Parent-Child Dyads: A Cross-Sectional Study. BMC Public Health.

[105] Whiting S., Buoncristiano M., Gelius P., Abu-Omar K., Pattison M., Hyska J., Duleva V., Musić Milanović S., Zamrazilová H., Hejgaard T. (2020). Physical Activity, Screen Time, and Sleep Duration of Children Aged 6–9 Years in 25 Countries: An Analysis within the WHO European Childhood Obesity Surveillance Initiative (COSI) 2015–2017. Obes. Facts.

[106] Fuemmeler B.F., Anderson C.B., Mâsse L.C. (2011). Parent-Child Relationship of Directly Measured Physical Activity. Int. J. Behav. Nutr. Phys. Act..

[107] Hinkley T., Crawford D., Salmon J., Okely A.D., Hesketh K. (2008). Preschool Children and Physical Activity. Am. J. Prev. Med..

[108] Nettle H., Sprogis E. (2011). Pediatric Exercise: Truth and/or Consequences. Sports Med. Arthrosc. Rev..

[109] Haverly K., Davison K.K. (2005). Personal Fulfillment Motivates Adolescents to Be Physically Active. Arch. Pediatr. Adolesc. Med..

[110] Trost S.G., Pate R.R., Ward D.S., Saunders R., Riner W. (1999). Correlates of Objectively Measured Physical Activity in Preadolescent Youth. Am. J. Prev. Med..

[111] Riddoch C.J., Bo Andersen L., Wedderkopp N., Harro M., Klasson-Heggebø L., Sardinha L.B., Cooper A.R., Ekelund U. (2004). Physical Activity Levels and Patterns of 9- and 15-Yr-Old European Children. Med. Sci. Sports Exerc..

[112] Garaulet M., Martínez A., Victoria F., Pérez–Llamas F., Ortega R.M., Zamora S. (2000). Differences in Dietary Intake and Activity Level Between Normal-Weight and Overweight or Obese Adolescents. J. Pediatric Gastroenterol. Nutr..

[113] Raistenskis J., Sidlauskiene A., Strukcinskiene B., Uğur Baysal S., Buckus R. (2016). Physical Activity and Physical Fitness in Obese, Overweight, and Normal-Weight Children. Turk. J. Med. Sci..

[114] Lovecchio N., Zago M. (2019). Fitness Differences According to BMI Categories: A New Point of View. J. Sports Med. Phys. Fitness.

[115] Yu J.J., Capio C.M., Abernethy B., Sit C.H.P. (2021). Moderate-to-Vigorous Physical Activity and Sedentary Behavior in Children with and without Developmental Coordination Disorder: Associations with Fundamental Movement Skills. Res. Dev. Disabil..

[116] Babic M.J., Morgan P.J., Plotnikoff R.C., Lonsdale C., White R.L., Lubans D.R. (2014). Physical Activity and Physical Self-Concept in Youth: Systematic Review and Meta-Analysis. Sports Med..

[117] Stodden D.F., Goodway J.D., Langendorfer S.J., Roberton M.A., Rudisill M.E., Garcia C., Garcia L.E. (2008). A Developmental Perspective on the Role of Motor Skill Competence in Physical Activity: An Emergent Relationship. Quest.

[118] Vandoni M., Calcaterra V., Carnevale Pellino V., De Silvestri A., Marin L., Zuccotti G.V., Tranfaglia V., Giuriato M., Codella R., Lovecchio N. (2021). “Fitness and Fatness” in Children and Adolescents: An Italian Cross-Sectional Study. Children.

[119] Booth F.W., Roberts C.K., Laye M.J., Terjung R. (2012). Lack of Exercise Is a Major Cause of Chronic Diseases. Comprehensive Physiology.

[120] Nimmo M.A., Leggate M., Viana J.L., King J.A. (2013). The Effect of Physical Activity on Mediators of Inflammation. Diabetes Obes. Metab..

[121] Beavers K.M., Brinkley T.E., Nicklas B.J. (2010). Effect of Exercise Training on Chronic Inflammation. Clin. Chim. Acta.

[122] Bruunsgaard H. (2005). Physical Activity and Modulation of Systemic Low-Level Inflammation. J. Leukoc. Biol..

[123] Aadland E., Kvalheim O.M., Hansen B.H., Kriemler S., Ried-Larsen M., Wedderkopp N., Sardinha L.B., Møller N.C., Hallal P.C., Anderssen S.A. (2020). The Multivariate Physical Activity Signature Associated with Metabolic Health in Children and Youth: An International Children’s Accelerometry Database (ICAD) Analysis. Prev. Med..

[124] Ekelund U. (2012). Moderate to Vigorous Physical Activity and Sedentary Time and Cardiometabolic Risk Factors in Children and Adolescents. JAMA.

[125] Haapala E.A., Väistö J., Ihalainen J.K., Tomaselli González C., Leppänen M.H., Veijalainen A., Sallinen T., Eloranta A.-M., Ekelund U., Schwab U. (2021). Associations of Physical Activity, Sedentary Time, and Diet Quality with Biomarkers of Inflammation in Children. Eur. J. Sport Sci..

[126] Lavie C.J., Church T.S., Milani R.V., Earnest C.P. (2011). Impact of Physical Activity, Cardiorespiratory Fitness, and Exercise Training on Markers of Inflammation. J. Cardiopulm. Rehabil. Prev..

[127] Fedewa M.V., Hathaway E.D., Ward-Ritacco C.L. (2017). Effect of Exercise Training on C Reactive Protein: A Systematic Review and Meta-Analysis of Randomised and Non-Randomised Controlled Trials. Br. J. Sports Med..

[128] Kasapis C., Thompson P.D. (2005). The Effects of Physical Activity on Serum C-Reactive Protein and Inflammatory Markers. J. Am. Coll. Cardiol..

[129] Pedersen B.K., Febbraio M.A. (2012). Muscles, Exercise and Obesity: Skeletal Muscle as a Secretory Organ. Nat. Rev. Endocrinol..

[130] Pedersen B.K., Febbraio M.A. (2008). Muscle as an Endocrine Organ: Focus on Muscle-Derived Interleukin-6. Physiol. Rev..

[131] Brandt C., Pedersen B.K. (2010). The Role of Exercise-Induced Myokines in Muscle Homeostasis and the Defense against Chronic Diseases. J. Biomed. Biotechnol..

[132] Fischer C.P. (2006). Interleukin-6 in Acute Exercise and Training: What Is the Biological Relevance?. Exerc. Immunol. Rev..

[133] Pedersen B.K. (2012). Muscular Interleukin-6 and Its Role as an Energy Sensor. Med. Sci. Sports Exerc..

[134] Petersen A.M.W., Pedersen B.K. (2005). The Anti-Inflammatory Effect of Exercise. J. Appl. Physiol..

[135] Starkie R., Ostrowski S.R., Jauffred S., Febbraio M., Pedersen B.K. (2003). Exercise and IL-6 Infusion Inhibit Endotoxin-induced TNF-α Production in Humans. FASEB J..

[136] Bergmann M., Gornikiewicz A., Sautner T., Waldmann E., Weber T., Mittlböck M., Roth E., Függer R. (1999). Attenuation of Catecholamine-Induced Immunosuppression in Whole Blood from Patients with Sepsis. Shock.

[137] Steensberg A., Fischer C.P., Keller C., Møller K., Pedersen B.K. (2003). IL-6 Enhances Plasma IL-1ra, IL-10, and Cortisol in Humans. Am. J. Physiol. Endocrinol. Metab..

[138] Carbó N., López-Soriano J., Costelli P., Alvarez B., Busquets S., Baccino F.M., Quinn L.S., López-Soriano F.J., Argilés J.M. (2001). Interleukin-15 Mediates Reciprocal Regulation of Adipose and Muscle Mass: A Potential Role in Body Weight Control. Biochim. Et Biophys. Acta (BBA)—Gen. Subj..

[139] Nielsen A.R., Hojman P., Erikstrup C., Fischer C.P., Plomgaard P., Mounier R., Mortensen O.H., Broholm C., Taudorf S., Krogh-Madsen R. (2008). Association between Interleukin-15 and Obesity: Interleukin-15 as a Potential Regulator of Fat Mass. J. Clin. Endocrinol. Metab..

[140] Walsh N.P., Gleeson M., Shephard R.J., Gleeson M., Woods J.A., Bishop N.C., Fleshner M., Green C., Pedersen B.K., Hoffman-Goetz L. (2011). Position Statement. Part One: Immune Function and Exercise. Exerc. Immunol. Rev..

[141] Sirico F., Bianco A., D’Alicandro G., Castaldo C., Montagnani S., Spera R., Di Meglio F., Nurzynska D. (2018). Effects of Physical Exercise on Adiponectin, Leptin, and Inflammatory Markers in Childhood Obesity: Systematic Review and Meta-Analysis. Child. Obes..

[142] Tenório T.R.S., Balagopal P.B., Andersen L.B., Ritti-Dias R.M., Hill J.O., Lofrano-Prado M.C., Prado W.L. (2018). Effect of Low- Versus High-Intensity Exercise Training on Biomarkers of Inflammation and Endothelial Dysfunction in Adolescents With Obesity: A 6-Month Randomized Exercise Intervention Study. Pediatric Exerc. Sci..

[143] Merlin M., de Oliveira H.H., Passos M.E.P., Momesso C.M., dos Santos de Oliveira L.C., Santana J.E., Levada-Pires A.C., Hatanaka E., Massao-Hirabara S., Guaré R. (2021). Relationship between Children Physical Activity, Inflammatory Mediators and Lymphocyte Activation: Possible Impact of Social Isolation (COVID-19). Sport Sci. Health.

[144] Stewart L.K., Flynn M.G., Campbell W.W., Craig B.A., Robinson J.P., McFarlin B.K., Timmerman K.L., Coen P.M., Felker J., Talbert E. (2005). Influence of Exercise Training and Age on CD14+ Cell-Surface Expression of Toll-like Receptor 2 and 4. Brain Behav. Immun..

[145] Da Luz Scheffer D., Latini A. (2020). Exercise-Induced Immune System Response: Anti-Inflammatory Status on Peripheral and Central Organs. Biochim. Biophys. Acta (BBA)-Mol. Basis Dis..

[146] Wang J., Liu S., Li G., Xiao J., Xiao J. (2020). Exercise Regulates the Immune System. Physical Exercise for Human Health.

[147] Hojman P. (2017). Exercise Protects from Cancer through Regulation of Immune Function and Inflammation. Biochem. Soc. Trans..

[148] Lancaster G.I., Febbraio M.A. (2014). The Immunomodulating Role of Exercise in Metabolic Disease. Trends Immunol..

[149] Apostolopoulos V., Borkoles E., Polman R., Stojanovska L. (2014). Physical and Immunological Aspects of Exercise in Chronic Diseases. Immunotherapy.

[150] Mach N., Fuster-Botella D. (2017). Endurance Exercise and Gut Microbiota: A Review. J. Sport Health Sci..

[151] Asimakos A., Toumpanakis D., Karatza M.-H., Vasileiou S., Katsaounou P., Mastora Z., Vassilakopoulos T. (2018). Immune Cell Response to Strenuous Resistive Breathing: Comparison with Whole Body Exercise and the Effects of Antioxidants. Int. J. Chronic Obstr. Pulm. Dis..

[152] Wang L., Lv Y., Li G., Xiao J. (2018). MicroRNAs in Heart and Circulation during Physical Exercise. J. Sport Health Sci..

[153] Freidenreich D.J., Volek J.S. (2012). Immune Responses to Resistance Exercise. Exerc. Immunol. Rev..

[154] Bigley A.B., Simpson R.J. (2015). NK Cells and Exercise: Implications for Cancer Immunotherapy and Survivorship. Discov. Med..

[155] Simpson R.J., McFarlin B.K., McSporran C., Spielmann G., ó Hartaigh B., Guy K. (2009). Toll-like Receptor Expression on Classic and pro-Inflammatory Blood Monocytes after Acute Exercise in Humans. Brain Behav. Immun..

[156] Deckx N., Wens I., Nuyts A.H., Lee W.-P., Hens N., Koppen G., Goossens H., Van Damme P., Berneman Z.N., Eijnde B.O. (2015). Rapid Exercise-Induced Mobilization of Dendritic Cells Is Potentially Mediated by a Flt3L- and MMP-9-Dependent Process in Multiple Sclerosis. Mediat. Inflamm..

[157] Timmons B.W., Cieslak T. (2008). Human Natural Killer Cell Subsets and Acute Exercise: A Brief Review. Exerc. Immunol. Rev..

[158] Janeway C., Murphy K.P., Travers P., Walport M. (2008). Janeway’s Immuno Biology.

[159] Peake J.M., Neubauer O., Walsh N.P., Simpson R.J. (2017). Recovery of the Immune System after Exercise. J. Appl. Physiol..

[160] Thomas N.E., Williams D.R.R. (2008). Inflammatory Factors, Physical Activity, and Physical Fitness in Young People: Inflammatory Factors, Activity, and Fitness in Young People. Scand. J. Med. Sci. Sports.

[161] Plaisance E.P., Grandjean P.W. (2006). Physical Activity and High-Sensitivity C-Reactive Protein. Sports Med..

[162] Wärnberg J., Nova E., Moreno L.A., Romeo J., Mesana M.I., Ruiz J.R., Ortega F.B., Sjöström M., Bueno M., AVENA Study group (2006). Inflammatory Proteins Are Related to Total and Abdominal Adiposity in a Healthy Adolescent Population: The AVENA Study. Am. J. Clin. Nutr..

[163] Wärnberg J., Moreno L.A., Mesana M.I., Marcos A., the AVENA group (2004). Inflammatory Mediators in Overweight and Obese Spanish Adolescents. The AVENA Study. Int. J. Obes..

[164] Trayhurn P., Wood I.S. (2004). Adipokines: Inflammation and the Pleiotropic Role of White Adipose Tissue. Br. J. Nutr..

[165] Ruiz J.R., Ortega F.B., Warnberg J., Sjöström M. (2007). Associations of Low-Grade Inflammation with Physical Activity, Fitness and Fatness in Prepubertal Children; the European Youth Heart Study. Int. J. Obes..

[166] Ruiz J.R., Rizzo N.S., Hurtig-Wennlöf A., Ortega F.B., Wàrnberg J., Sjöström M. (2006). Relations of Total Physical Activity and Intensity to Fitness and Fatness in Children: The European Youth Heart Study. Am. J. Clin. Nutr..

[167] Gutin B., Yin Z., Humphries M.C., Barbeau P. (2005). Relations of Moderate and Vigorous Physical Activity to Fitness and Fatness in Adolescents. Am. J. Clin. Nutr..

[168] Platat C., Wagner A., Klumpp T., Schweitzer B., Simon C. (2006). Relationships of Physical Activity with Metabolic Syndrome Features and Low-Grade Inflammation in Adolescents. Diabetologia.

[169] Cook D.G., Mendall M.A., Whincup P.H., Carey I.M., Ballam L., Morris J.E., Miller G.J., Strachan D.P. (2000). C-Reactive Protein Concentration in Children: Relationship to Adiposity and Other Cardiovascular Risk Factors. Atherosclerosis.

[170] Kohl H.W., Fulton J.E., Caspersen C.J. (2000). Assessment of Physical Activity among Children and Adolescents: A Review and Synthesis. Prev. Med..

[171] Rowlands A.V., Eston R.G. (2007). The Measurement and Interpretation of Children’s Physical Activity. J. Sports Sci. Med..

[172] Han Y., Liu Y., Zhao Z., Zhen S., Chen J., Ding N., Ma Y., Wen D. (2019). Does Physical Activity-Based Intervention Improve Systemic Proinflammatory Cytokine Levels in Overweight or Obese Children and Adolescents? Insights from a Meta-Analysis of Randomized Control Trials. Obes. Facts.

